# Dispersal of Pleistocene *Equus* (Family Equidae) into South America and Calibration of GABI 3 Based on Evidence from Tarija, Bolivia

**DOI:** 10.1371/journal.pone.0059277

**Published:** 2013-03-20

**Authors:** Bruce J. MacFadden

**Affiliations:** Florida Museum of Natural History, University of Florida, Gainesville, Florida, United States of America; Raymond M. Alf Museum of Paleontology, United States of America

## Abstract

The dispersal of *Equus* into South America during the Great American Biotic Interchange (GABI) represented a major event for Pleistocene land-mammal age chronology on that continent. It has been argued that this dispersal occurred during the late Pleistocene, ∼0.125 Ma, and it defines the base of the Lujanian South American Land Mammal Age (SALMA). In this scenario, *Equus* dispersed during the fourth and latest recognized phase of the interchange, i.e., GABI 4. Although *Equus* was widely distributed in South America during the Pleistocene, only a few localities are calibrated by independent chronostratigraphic data. In this paper, new biostratigraphic evidence documents that *Equus* occurs from 15 superposed faunal horizons or zones throughout the Tolomosa Formation at Tarija, Bolivia. This biostratigraphic sequence is independently calibrated to occur between ∼0.99 to <0.76 Ma during the middle Pleistocene Ensenadan SALMA and coincident with GABI 3, not GABI 4. Tarija remains the only well calibrated Ensenadan locality at which *Equus* is found. The new biostratigraphic data presented here are unambiguous and document the earlier (pre-Lujanian) occurrence of this genus in South America. The hypothesized dispersal of the genus *Equus* into South America at ∼0.125 Ma is no longer supportable in light of the new biostratigraphic evidence presented here. The new data from Tarija thus have continent-wide implications for the origins and biogeography of *Equus* in South America as well as the calibration of GABI 3.

## Introduction

**Figure 1:**
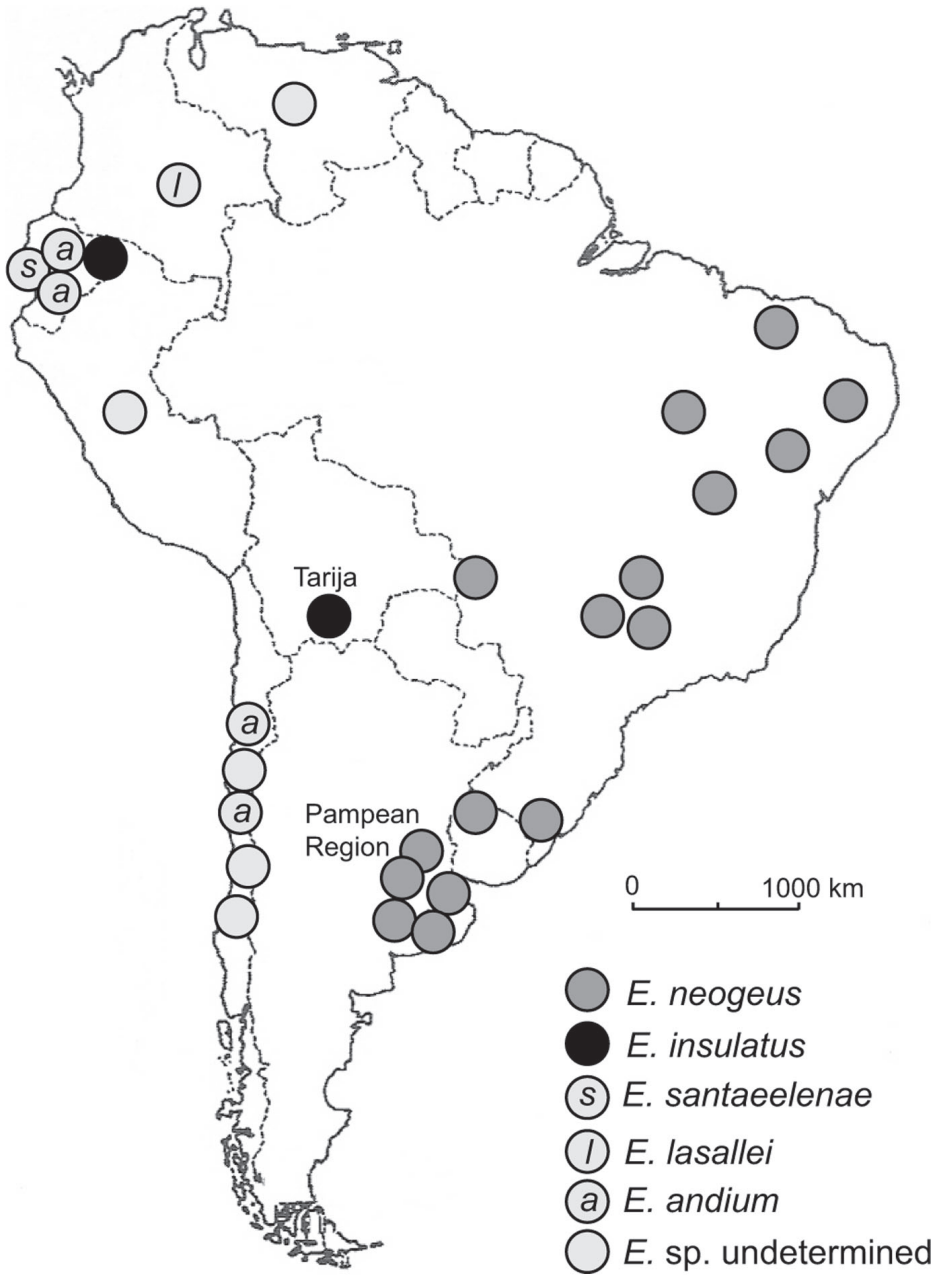
Map of *Equus* localities in South America. This map is modified, and includes all recognized species according to Alberdi and Prado [4]. Some points, e.g., *Equus* sp. from Colombia, actually represent multiple localities [5]. Of relevance to this paper, the species *E*. *insulatus* occurs from Tarija, Bolivia and one other locality, Rio Chiche, Ecuador [4]–[6]. The locality from Ecuador is reported to be late Pleistocene, but otherwise lacks precise, independent chronostratigraphic control.

The dispersal of the horse *Equus* into South America during the Great American Biotic Interchange (GABI) represents an important event in the historical biogeography of Pleistocene mammals on that continent. Several recent studies have asserted that the age of this dispersal event is late Pleistocene, ∼0.125 Ma, during a late phase of the GABI, i.e., GABI 4 [1]–[3]. This event takes on further significance because it defines the base of, and is an index fossil for, the Lujanian South American Land Mammal Age (SALMA) as it is characterized from classic outcrops in the Pampean region of Argentina. Five currently recognized extinct species of *Equus* [4] are found at numerous localities through South America (Figure 1). So far as the biochronology is known, most of these occurrences are late Pleistocene [5], [6], with one significant exception from the Tarija basin of Bolivia. The geochronology and biostratigraphy of the fossil mammals at Tarija have recently been called into question by some workers [7], [8].

Given the importance of the Tarija equids, and in particular the biochronology of *Equus* in South America, the purpose of this paper is to: (1) document the precise biostratigraphic occurrence of the three equid species, *E. insulatus*, *Hippidion principale,* and *Onohippidium devillei;* and (2) discuss the broader importance of these occurrences with regard to currently accepted SALMA chronology, particularly as compared to the classic sequences in the Pampean region of Argentina. As we will see below, the results also have significant ramifications for an understanding of the overall paleobiogeography of *Equus* in South America. For example, was there one dispersal event, or were there multiple dispersals, of this genus into South America during the GABI?

The sediments deposited within the Tarija Basin, southern Bolivia, contain a rich mammalian fauna of middle Pleistocene age [9]. The Tarija Fauna serves as an important basis for understanding South American Land Mammal Age (SALMA) biochronology as compared to the classic stratigraphic sections and mammalian faunal evolution represented in the Pampean region of Argentina. The specific biochronological age of the Tarija Fauna is typically stated to be Ensenadan [4], [5], [9], [10], although the possibility that some of this fauna is post-Ensenadan (i.e., either Bonaerian or Lujanian SALMA) cannot be ruled out [8]. In a radical departure from the accepted norm, based on supposedly in situ radiocarbon ages, Coltorti et al. [7] concluded that the entire Tolomosa Formation at Tarija is latest Pleistocene in age (<60,000 years); nevertheless, MacFadden et al. [11] present new data that refute the Coltorti et al. [7] interpretation and further confirm the middle Pleistocene age for the fossiliferous sequence at Tarija.

In order to resolve the biochronological age(s) of the Tarija Fauna, we need to know if post-Ensenadan taxa, i.e., as these SALMA biochrons are typified in Argentina, occur stratigraphically above, and in demonstrable superposition with, those of Ensenadan age. Despite the fact that tens of thousands of fossils have been collected from Tarija over the past six centuries, almost none of these collections contain precise stratigraphic information that would resolve the temporal sequence and transition from Ensenadan to post-Ensenadan ages. More recent collections made, for example, by the Japanese mission in the 1970s and 1980s [12], [13] and field expeditions from the University of Florida (1978 to 1986) contain biostratigraphic data, and for the latter collection, a database of individual fossil occurrences. Nevertheless, these data sets have yet to be fully analyzed and documented in the literature. Realizing the importance of the Tarija biochronological framework, Tonni et al. [8] recently presented a preliminary biostratigraphic analysis based on examination of existing collections matched to three lithological members developed by Takai et al. [12], [13], as will be discussed below.

Fossil horses (Family Equidae) are a major component of the dispersal of northern immigrants into South America during the Great American Biotic Interchange (GABI). Following several authors [14]–[17], three equid genera and species occur in the Tarija Fauna (Tolomosa Formation), i.e., the hippidiforms *Hippidion principale* and *Onohippidium devillei*, and equine *Equus insulatus* [16], [17]. It should also be noted that some other workers [18] consider *Hippidion* Owen, 1869 and *Onohippidium* Moreno, 1891 to be congeneric, and thus the two hippidiform horses at Tarija have recently been referred to *H. principale* and *H. devillei*. Nevertheless, this latter interpretation is not followed here for a variety of reasons [17], [19]. This taxonomic nuance is irrelevant to the fact that three species of extinct equids exist at Tarija and their individual biochrons are important for understanding Pleistocene SALMAs, particularly as these are compared to the classic localities in the Pampean region of Argentina.

The timing of the dispersal events for the three equid genera *Hippidion, Onohippidium*, and *Equus* has received considerable attention in the literature, and yet these are still poorly constrained. Marshall et al. [5] stated that the hippidiform genera first occur in South America during the late Pliocene (Uquian SALMA), ∼2.5 Ma, whereas *Equus* dispersed into South America later, possibly 1.5 Ma (also [20]). This was based on very few localities with associated independent geochronological control, and as we will see below, the timing of the arrival of *Equus* in South America still remains problematical, despite its importance as a SALMA index fossil.

MacFadden [17] stated that *Equus* occurs throughout the stratigraphic sequence from Tarija, which has its lower limits based on associated magneto-and chronostratigraphy of ∼1 Ma [9], [11]. This occurrence poses a particular problem with regard to the biochronological framework for the SALMAs: Several recent studies posit that that *Equus* dispersed into South American during a late immigration pulse, i.e., GABI 4, arguing that this genus reached South America during the late Pleistocene, no earlier than ∼0.125 Ma [2], [3], [21]. For example, Cione and Tonni [2] argued that the presence of *Equus* in the Pampean region is exclusive to the Lujanian SALMA and is the index fossil that defines the *E. neogeus* biozone (also see [22]). Thus, so far as it has been dated, the only place in South America in which *Equus* is found earlier than the Lujanian is Tarija, although Tonni et al. ([8] p. 63) stated that “there are no descriptions of the specimens attributed to *Equus* from the lower part of the Tolomosa Formation.”

### Geological and Biostratigraphic Context of the Tolomosa Formation and Tarija Fauna

**Figure 2:**
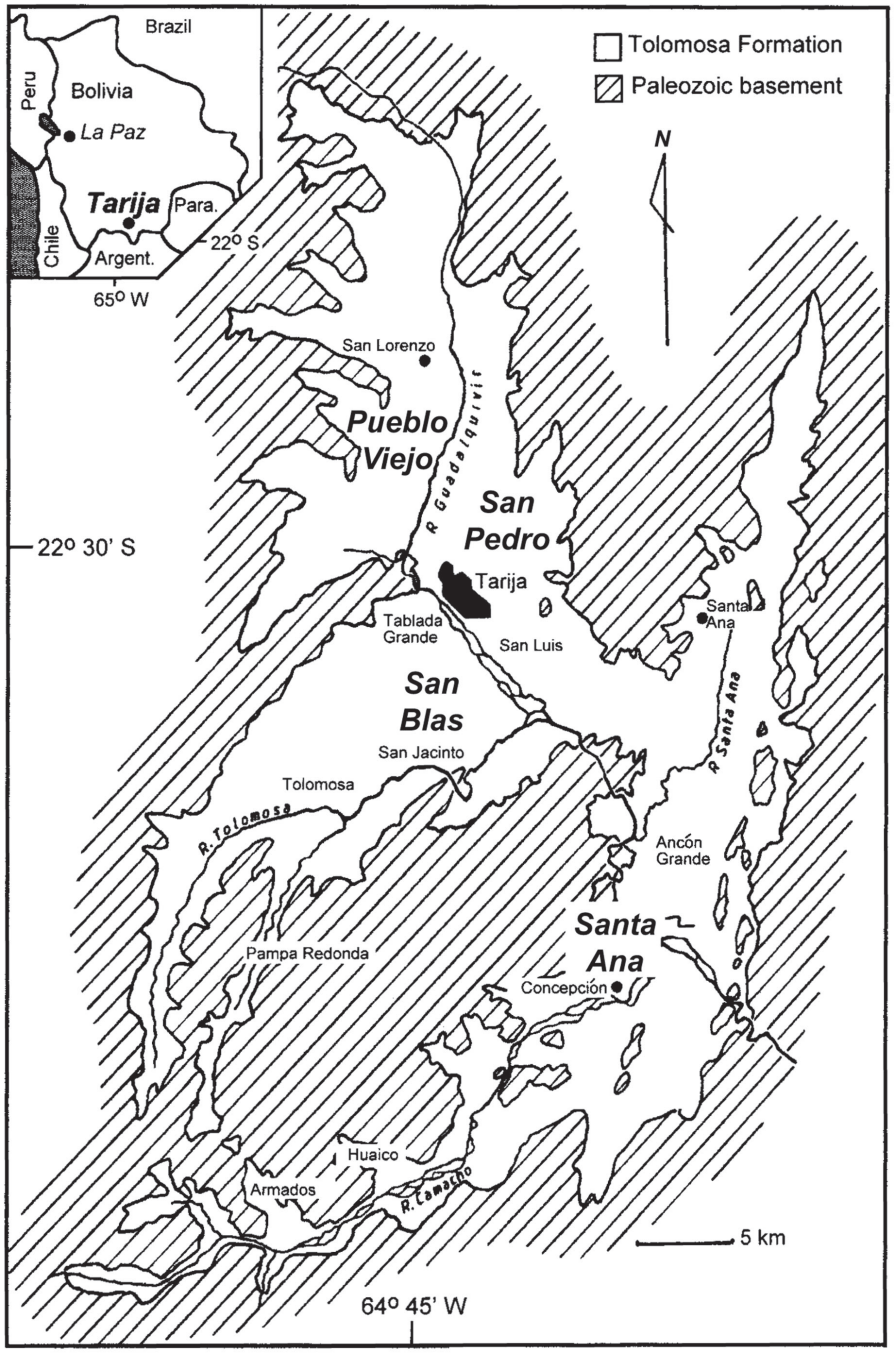
Geographic location of Tarija, Bolivia. Map locating Tarija (insert, upper left) in southern Bolivia and distribution of outcrops of the Tolomosa Formation surrounding the city of Tarija, with the location of the four measured sections discussed in this paper [8], [10], [24]. Also see correspondence with Figure 3.

**Figure 3:**
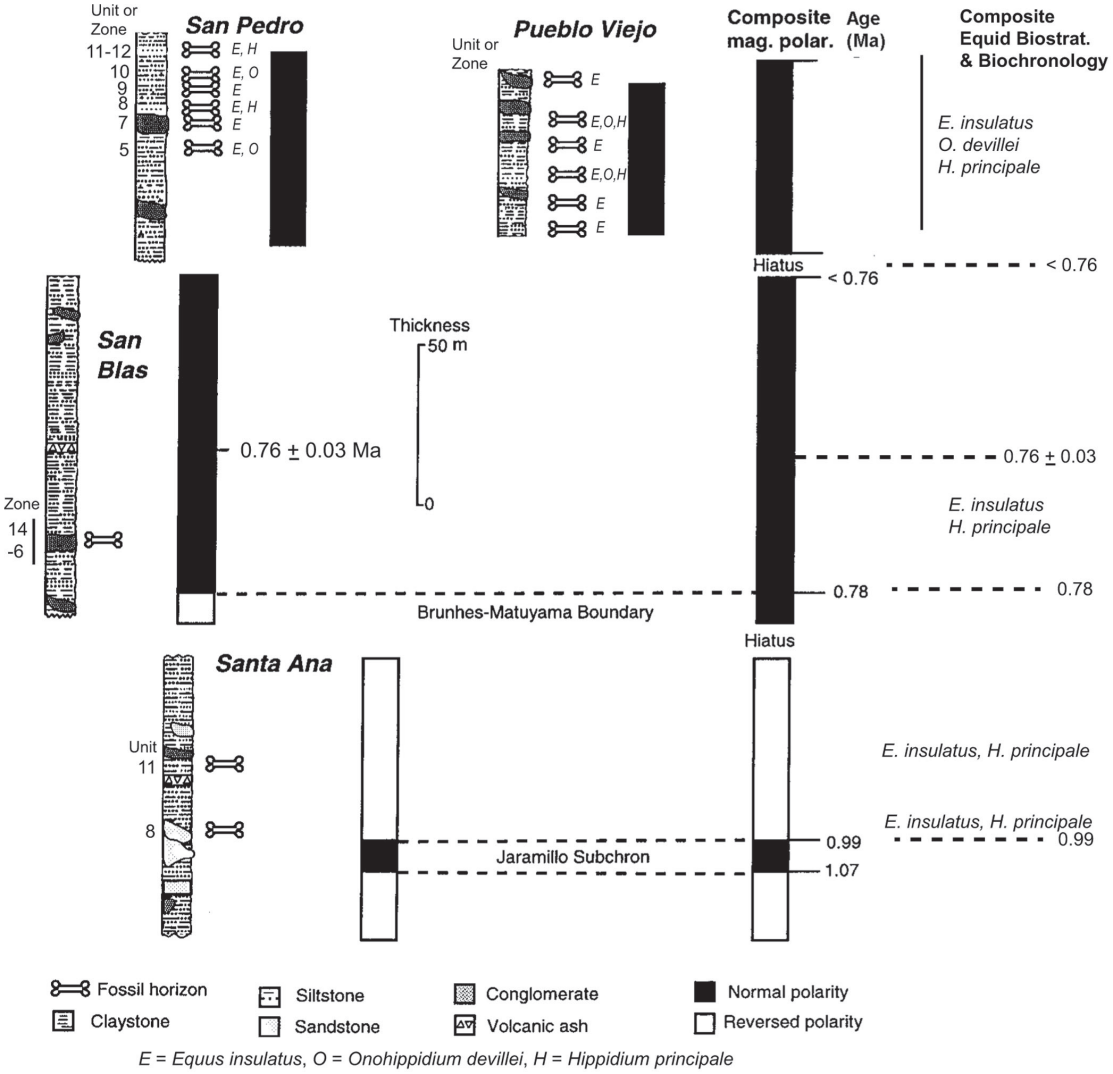
Composite section at Tarija and correlation to GPTS. Composite measured section, biostratigraphic levels showing distribution of the equids, ages, and correlation to the time-scale (GPTS) represented in the Tolomosa Formation, modified from [9]. The ages for the magnetostratigraphic boundaries follow Gee and Kent [25].

The fossiliferous sediments of the Tolomosa Formation crop out as badlands in a submontane Andean basin around the city of Tarija in southern Bolivia (approx. lat. 21° 30′ S, long. 65° 45′ W; Figure 2) at a general elevation of ∼1800 m. Oppenheim [23] presented a general description of the lithostratigraphy of these outcrops (which were previously referred to as the Tarija Formation) which include clays and silts, and lenticular, cross-bedded sands and conglomerates, with ferruginous zones and paleosol horizons. Several tuffaceous zones occur in the Tolomosa Formation, including the prominent San Blas ash that is a radioisotopic calibration point for the composite measured section [9], [11]. The dominant mode of deposition of these sediments was fluvial, with some possible aeolian influences resulting in loess. As shown in Figure 2, MacFadden et al. [9] measured four individual stratigraphic sections, i.e., Pueblo Viejo (21° 25′S, 64° 45′ W), San Blas (21° 35′ S, 64° 43′ W), Santa Ana (21° 31′S, 64° 39′ W), and San Pedro (21° 30′ S, 64° 41′ W). The composite measured section spans ∼280 m of the Tolomosa Formation (Figure 3). The magnetostratigraphy includes the Brunhes (C1n)-Matuyama boundary, which is currently calibrated to be 0.78 Ma [25], at 110 m above the base of the composite section presented in MacFadden et al. [9]. This section also is calibrated by a radioisotopic age of 0.76±0.03 Ma [11] for a prominent ash in the San Blas section that lies 40 m above the Brunhes-Matuyama boundary.

With regard to previous biostratigraphic frameworks for the mammals from Tarija, Takai [12], [13] divided the Tolomosa Formation into a tripartite scheme of lower, middle, and upper members. In contrast to MacFadden [17], Takai [12] indicated that *Equus* only occurs in the upper member of the Tolomosa Formation. Tonni et al. [8] followed the tripartite scheme proposed by Takai [12], and seemed to accept the hypothesis that *Equus* only occurs in the upper member. With regard to biochronology, i.e., unconstrained by biostratigraphy, Tonni et al. [8] analyzed the entire Tarija Fauna as it is compared to individual taxonomic ranges in the Pampean region. They conclude that the overall Tarija Fauna mixes faunal elements, i.e., some of which are Ensenadan, whereas others are either Bonaerian or Lujanian. *Equus* from Tarija (as *E. insulatus*) is purported to indicate a Lujanian biochron.

## Materials and Methods

### Ethics Statement

No permits were required for the described study, which complied with all relevant regulations.

### Biostratigraphy

The biostratigraphic data used during this study were retrieved from the UF Vertebrate Paleontology collections database [26]. All specimens included in the database (Table S1) were retrieved using ‘Tarija’, ‘Equidae’, and occur from one of four measured sections [9], i.e., Pueblo Viejo, San Blas, Santa Ana, and San Pedro (Figure 3). The entire Tarija equid database consists of 380 catalogued specimens with known stratigraphic provenience. Of this collection, 297 are identified to genus and species (the other specimens mostly represent fragmentary and/or postcranial materials that are not currently identified to genus and species) and comprise the database used in this analysis. The taxonomic identifications of these specimens were confirmed by visual inspection during this study. The occurrences of each of the three equid species at the 15 fossiliferous units or zones were plotted on the measured sections (Figure 3).

### Dental Measurements and Statistics

Dental measurements were taken for all available specimens of *Equus insulatus* in the UF collection that had either an upper first (M1) or second (M2) molar and known biostratigraphic provenience. The measurements were taken on all available specimens, and presented in Table S1 as follows: (1) M12APL, greatest anterior-posterior length of enamel (excluding cement) of either an M1 or M2 at the occlusal surface; (2) M12TRN, greatest transverse width of enamel (excluding cement) of either an M1 or M2 at the occlusal surface; (3) M12CRNHT, mesostyle crown height of M1 or M2. Because of tooth wear related to ontogeny, each tooth that was measured was qualitatively coded as J, juvenile; A, adult; O, old age. The descriptive statistics and ANOVA were calculated using Excel®.

## Results and Discussion

### Biostratigraphy and Relative Abundance

Of the 297 specimens of Tarija equids studied, 274 represent *Equus insulatus*, 17 *Onohippidium devillei*, and 6 *Hippidion principale*. These were collected from 15 stratigraphic units, or zones, within the four measured sections [9] (Figure 3), and range in age from ∼0.99 to >0.76 Ma (Figure 3; Table 1). These occurrences are distributed as follows:

**Table 1:** Stratigraphic distribution, by number (N) of catalogued specimens (identified to species), of the three horses from Tarija by site and unit, or zone.

Section	Unit, or Zone	Age	N specimens	N specimens	N specimens
		(Ma)	*Equus insulatus*	*Onohippidium devillei*	*Hippidion principale*
Santa Ana	8	∼0.99	2	1	–
	11	<0.99, >0.78	3	9	–
San Blas	6–14	<0.78, >0.76	36	–	2
San Pedro	5	<0/78	2	1	–
	7	<0.78	23	–	–
	8	>0.78	2	–	1
	9	<0.78	45	–	–
	10	<0.78	5	2	–
	11–12	<0.78	4	–	1
Pueblo Viejo	1	<0.78	1	–	–
	5	<0.78	4	–	–
	7	<0.78	96	3	1
	8	<0.78	26	–	–
	9–10	<0.78	22	1	1
	12	<0.78	3	–	–
Totals		∼0.99 to<0.76	274	17	6
% represented			92.3	5.7	2.0

The raw data from which this table is compiled are presented in Table S1.

#### Santa ana

**Figure 4:**
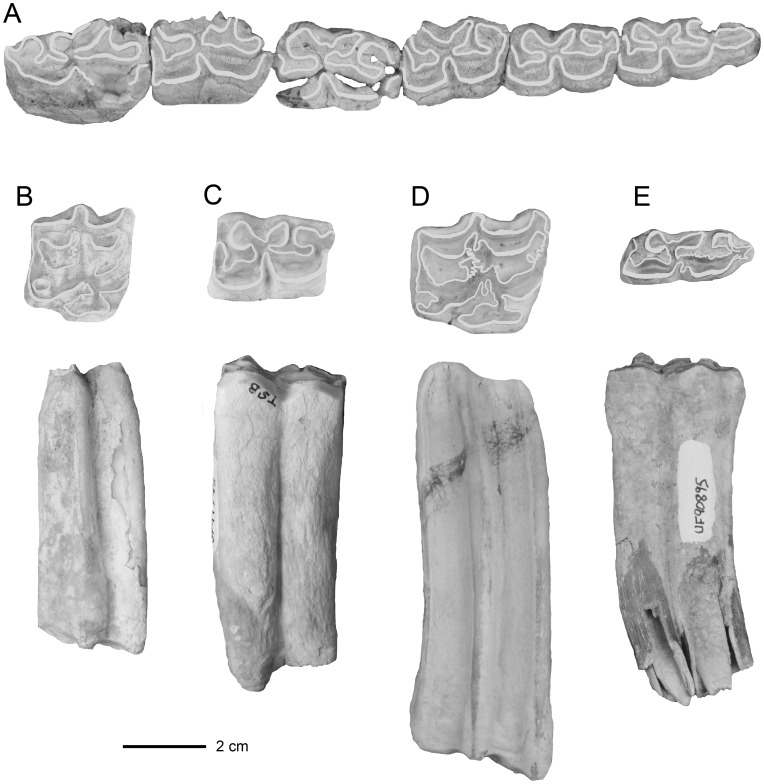
Dentitions of *Equus insulatus* from Tarija, Bolivia. Cheek teeth from different stratigraphic levels within the measured section of the Tolomosa Formation at Tarija (Figure 3). A. UF 91292, left cheek tooth dentition (p2-m3) from Santa Ana, unit 8. B. UF 92130, right M1 or M2, occlusal and lateral views, from Santa Ana, unit 8. C. UF 91795, left m1 or m2, occlusal and lateral views from San Blas, zone 6–14. D. UF 90890, right M2, occlusal and lateral views, from Pueblo Viejo, unit 10. E. UF 90895, left m1 or m2, occlusal and lateral views, from Pueblo Viejo, unit 10. The enamel outlines in the occlusal views are digitally enhanced for emphasis.

Two units in the middle of the section produce a total of 15 fossil equids from Santa Ana, i.e., 5 specimens of *E. insulatus* and 10 of *O. devillei* (Table 1). These were collected from unit 8 at 30 m and unit 11 at 50 m above the base of the measured section (Figure 3). Unit 8 approximates the top of the Jaramillo Subchron (C1r.1n) at 0.99 Ma [9], [11], and unit 11 is within the late part of the Matuyama Chron, which is therefore constrained between 0.99 and 0.78 Ma. A relatively well-preserved lower dentition (UF 91292, Figure 4A) and RM1 or M2 (UF 92130, Figure 4B) of *E. insulatus* clearly documents the characteristic equine dental morphology of this species (as opposed to the hippidiforms) in the lower (actually lowest) stratigraphic levels within the composite measured section of the Tolomosa Formation [9]. Relative to either *H. principale* or *O. devillei*, diagnostic characters seen in these specimens of *E. insulatus* include relative degree of hypsodonty (more high-crowned), complex enamel foldings in both the upper and lower teeth, elongated protocone with angular (rather than rounded) anterior and posterior borders and pli caballin in the uppers, and in the lowers, moderately shallow ectoflexids and widely flared and expanded metaconids and metastylids with angular anterior and posterior borders [5], [16], [17].

#### San blas

Relative to the other sections, the San Blas section is not particularly fossiliferous, except for a narrow zone encompassing units 6–14 between about 20–25 m above the base of the local measured section. The equids include 36 specimens of *E. insulatus* and 2 specimens of *H. principale*. These occurrences are very well constrained above the Brunhes-Matuyama boundary at 0.78 Ma [24] and below the San Blas ash, which has an age of 0.76±0.03 Ma [11].

#### San pedro

**Figure 5:**
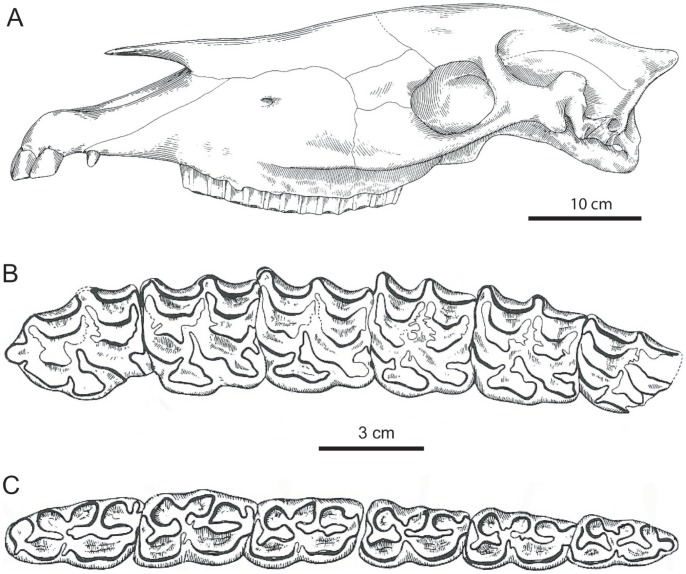
Cranium, mandible, and dentition of *Equus insulatus* from Tarija, Bolivia. UF 90551, from Tarija, San Pedro section, zone 11–12 (Figure 3). A. Left lateral view of cranium. B. left P2-M3. C. left p2-m3. Modified from MacFadden and Azzaroli [16].

The equids collected from seven units or zones within the upper part of the San Pedro measured section consist of 81 *E. insulatus*, 3 *O. devillei*, and 2 *H. principale* specimens. Based on the correlation of the composite section of the Tolomosa Formation to the GPTS, all of these occurrences of Tarija equids are constrained to be younger than the Brunhes-Matuyama boundary at 0.78 Ma. The best preserved specimen in the UF collections, consisting of a cranium and mandible of *E. insulatus*, comes from unit 11 near the top of the measured section [16] (Figure 5).

#### Pueblo viejo

More than half of the specimens of Tarija equids (158, or 53.2%) were collected from Pueblo Viejo. These come from six units or zones and, except for one specimen from near the base of the section (unit 1), are distributed within the upper three-quarters (50 m) of the measured section. Similar to San Blas and San Pedro, *E. insulatus* is abundant (152 specimens) relative to *O. devillei* (4 specimens), and *H. principale* (1 specimen). Within our collections, the three species of equids co-occur at unit 7 and zone 9–10 in Pueblo Viejo (Figure 3); this is the only instance in which all three species are found a single horizon or zone within our composite measured section of the Tolomosa Formation. Based on the correlation of the composite section of the Tolomosa Formation to the GPTS, these occurrences of Tarija equids from Pueblo Viejo are constrained to be younger than the Brunhes-Matuyama boundary at 0.78 Ma.

In summary, *Equus insulatus* is overwhelmingly abundant (274 of 297 specimens), representing 92.3% of the equids in the database analyzed here from our Tarija collections (Table S1). In addition, these specimens of *E. insulatus* occur throughout the known biostratigraphic sequence at Tarija and they are found at all 15 units or zones that contain fossil equids. *Onohippidium devillei* is rare, with 10 of the 16 specimens coming from the Santa Ana section and other specimens coming from San Pedro (3) and Pueblo Viejo (4). With only 6 specimens in our collection, *Hippidion principale* is very rare and only represented at four units or zones within three of our measured sections (San Blas, San Pedro and Pueblo Viejo); it is not found from the Santa Ana section. The absence of *H. principale* at Santa Ana, which is the oldest part of the Tarija sequence, is not considered significant because this species is reported to occur earlier elsewhere in South America [5]; its absence is interpreted to represent a sampling artifact. It is therefore likely that all three equids lived in the Tarija basin during the time of interval represented by the Tolomosa Formation. The discontinuous biostratigraphic occurrences within the measured sections likely represent incomplete sampling of the relatively rare hippidiforms.

Tonni et al. [8] questioned the presence of *E. insulatus* and its identification in the lower part of the Tarija faunal sequence. The results presented here address this concern because this species is now unequivocally documented to occur at all 15 faunal and biochronological levels spanning from ∼0.99 Ma to <0.76 Ma.

### Dental and Size Variation of *Equus insulatus* throughout the Composite Section

The single equine species from Tarija has variously been described as, *E. andium* race *insulatus* [14], *E*. (*Amerhippus*) *insulatus* [4], [15], and *Equus insulatus* [16]. In the most recent comprehensive analysis of fossil *Equus* in South America, Alberdi and Prado [4] recognized at least 5 distinct species, including *E. neogeus*, *E. lasallei*, *E. insulatus*, *E. santaeelenae*, and *E. andium*.

Given the previously poor biostratigraphic resolution, this study investigated if there was any discernible morphological change in the size of *E. insulatus*, as well as rule out the occurrence of more than one species, as it (they) occur(s) throughout the ∼240,000+ year interval within the Tolomosa Formation. This interval includes significant cycles of climate change, including the interval between Marine Isotope Stages 29 through 17 or 15 [24]. It would therefore be interesting to see if there is any correlation between the climate cycles and morphology of E. *insulatus* represented in this sequence.

**Figure 6:**
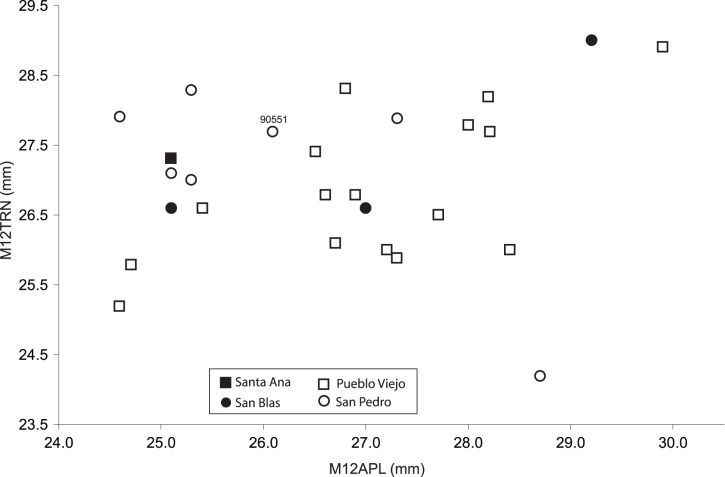
Dental measurements of *Equus insulatus* throughout the Tolomosa Formation. Plot of M12APL (anteroposterior length of upper M1 or M2) versus M12TRN (transverse width of upper M1 or M2) of all available specimens of *Equus insulatus* with associated biostratigraphic data from the Tarija Fauna, by locality. The symbol 90551 refers to the relevant measurements of the skull and mandible (UF 90551) of *Equus insulatus* from San Pedro (Figure 5). ANOVA does not find significant differences among the fossiliferous units or zones depicted in Figure 3.

The M12APL and M12TRN, which also serve as proxies for size variation [27], are plotted in Figure 6. ANOVA indicates that there are no statistical differences in these dental measurements from San Blas, Pueblo Viejo, and Pueblo Viejo (Table 2; the single relevant tooth from Santa Ana was excluded from the ANOVA, but is graphically represented in Figure 6). Both the M12APL and M12TRN appear to indicate a homogeneous pooled sample throughout the section. Likewise, the M12CRNHT (Old wear stage removed) is homogeneous (Table 2), indicating no apparent trends in hypsodonty within the section. Likewise, the Coefficients of Variation (*V*) for M12APL and M12TRN, of respectively, 5.6% and 4.3% are within the range that would be expected of a normal population or paleopopulation of, respectively, extant or extinct *Equus* [28]. The specific identification of *E. insulatus* from the lower part of the Tolomosa Formation, i.e., the Santa Ana and San Blas sections, is of further significance because both of these localities are demonstrably older than the Brunhes-Matuyama boundary at 0.78 Ma. Furthermore, the fossils from San Blas are in the direct superpositional sequence that includes the radiometrically dated San Blas ash at 0.76±0.03 Ma [11].

**Table 2:** Descriptive statistics and ANOVA for the three dental characters measured from the San Blas, San Pedro, and Pueblo Viejo sections from Tarija.*

Character	N**	Mean (mm)	STD (mm)	*V* (%)	F	F_crit_	*p*
M12APL	26	26.8	1.49	5.6	1.32	3.42	0.3153
M12TRN	26	27.0	1.15	4.3	0.32	3.42	0.7290
M12CRNHT	22	73.2	8.57	11.7	1.25	3.52	0.3020

The individual specimen measurements are presented in Table S1.

* Measurements for the one relevant specimen from Santa Ana, UF 92130, which is not used in the ANOVA (and therefore not presented in these descriptive statistics) is presented in Table S1.

** for M12APL and M1TRN, N by locality is San Blas (3), San Pedro (7), and Pueblo Viejo (16); for M12CRNHT, the N by locality is San Blas (5), San Pedro (5), and Pueblo Viejo (12).

In summary, so far as can be determined, only one species of *Equus* occurs within the Tarija faunal sequence and based on previous work, it is allocated to *E. insulatus*. While the sample sizes for this analysis appear to have been sufficient for the statistical comparisons, it is not prudent to speculate about evolutionary mode without a more complete suite of larger samples from individual fossil zones or horizons. In view of the marine isotope stages documented at Tarija [24], an investigation of microevolutionary change in *Equus* during known intervals of climate cycles would be interesting if larger, stratigraphically well-constrained samples could be assembled in the future.

## General Discussion

### GABI 3

Since the middle of the 20^th^ century, the Great American Biotic Interchange (GABI) has been considered to be a series of bilateral dispersal and immigration events between the Americas during the Pliocene and Pleistocene, which Simpson [29] referred to as the “Third Phase” (in reference to the two earlier phases during the Cenozoic that were not part of the GABI) that resulted from the formation of the land bridge in Panama. Refinements in calibrating the fossil record and additional discoveries since that time have further elucidated our understanding of the sequence of dispersal events within the GABI [30]. Woodburne [3] described these principal phases as GABI 1 (2.6 to 2.4 Ma), GABI 2 (∼1.8 Ma), GABI 3 (∼1.0 to 0.8 Ma) and GABI 4 (0.125 Ma). In his scenario, *Equus* dispersed into South America during GABI 4 and this event defines the base of the Lujanian SALMA (also see below). In contrast to this interpretation, the bio- and chronostratigraphic framework presented here for Tarija indicates that the first appearance of *Equus* in South America was part of GABI 3, not GABI 4.

As presented by Woodburne [3], GABI 3 also includes three other families of mammals that dispersed into South America, i.e., Mustelidae, Cervidae, and Tayassuidae, all three of which have been reported from Tarija [8], [14], [15]. Following more recent taxonomic assignments, of these three families from Tarija, the Mustelidae is represented by *Conepatus chinga*, Cervidae by *Hippocamelus* sp., and Tayassuidae by *Catagonus stenocephalus* and *Platygonus* sp. [8], [31]. As is characteristic of the majority of Tarija fossils, the biostratigraphic documentation of these taxa is poor; no fossils with known provenience representing these three families are contained in the UF collection. Frailey et al. [32] described *Hippocamelus* sp. from Pueblo Viejo (Figure 3), which is equivalent to the section that we measured [9]. Although we do not know the exact horizon from which it was collected, based on the magnetostratigraphy, this occurrence of *Hippocamelus* sp. equates to the early part of the Brunhes during the middle Pleistocene at <0.78 Ma. This however, must not be viewed as the age of the dispersal event. *Hippocamelus* sp. is rare at Tarija, and without additional biostratigraphic data, it is conceivable that it actually occurs earlier, but it is just not documented in the lower part of the section.

At the current level of biochronological and biostratigraphic resolution provided at Tarija, GABI 3 can be considered as a pulse in which four families of mammals dispersed into South American, and as Woodburne [3] posits, between ∼1.0 to 0.8 Ma. Teasing apart this event into more highly resolved dispersals of individual families would require a level of biochronological resolution not provided by the available fossil record.

### *Equus* as an Index Fossil in South America

The unequivocal presence of *Equus* at Tarija starting at 0.99 Ma calls into question the use of this genus as an index fossil for the late Pleistocene Lujanian SALMA, i.e., <0.125 Ma. Nevertheless, if *Equus* is restricted to the Pampean species *E. neogeus*, then the use of this latter taxon still can be used as an index fossil for the Lujanian within the Pampean region, and likely from other areas in which this species has been reported to occur, i.e., elsewhere in Argentina, as well as Uruguay and Brazil (Figure 1). Since *E. neogeus* is not recognized in the higher elevation regions elsewhere in South America, the use of this species therefore must be biogeographically restricted to where it is known to occur. GABI 4 includes several other families of mammals, including, the procyonid *Nasua*, mustelid *Lutra*, canid *Canis*, felid *Leopardus*, leporid *Sylvilagus*, and glyptodont *Glyptotherium* [3]. These other Lujanian taxa, which vary in their relative abundance and/or are quite rare at South American fossil sites, must also be reassessed for their biochronological ranges outside of the classic Pampean region. With a dearth of well dated late Pleistocene localities in South America, a reassessment of the utility of the Lujanian SALMA will continue to be a challenge.

### Biogeography and Paleoecology

Three species of equids dispersed into South America during the GABI. So far as can be determined, the more primitive hippidiforms *H. principale* and *O. devillei* dispersed into South America during the late Pliocene Vorohuean ( = Uquian of earlier authors, [5], [10], [30]) SALMA, apparently during GABI 1 [3]. At Tarija all three equids co-occur within the sequence, although the hippidiforms *H. principale* and *O. devillei* are, respectively, rare and very rare. It is perhaps tempting to speculate that competition occurred between the hippidiforms and *E. insulatus*, but they are quite different in body size and diet [17], [33]. Based on palynological evidence, Yoshida and Yamazaki [34] reconstruct the paleoenvironment at Tarija during the Pleistocene to be a dry, open-county grassland with scattered trees and shrubs mostly concentrated along the margins of rivers and lakes. MacFadden and Shockey [33] report a mean δ^13^C value of −3.8‰ (N = 9) for *E. insulatus*, indicating a diet of predominantly C4 grasses. Thus, the preponderance of *E. insulatus* at Tarija is best explained as a relatively specialized adaptation to grazing, unlike the other two sympatric hippidiform horses, both of which were more mixed feeders [33].

The presence of *E. insulatus* in the Ensenadan sequence at Tarija is problematical relative to its later occurrence in the classic sequence in Argentina. Tonni et al. [8] suggested a paleoecological or biogeographic explanation for this temporal disparity. If one looks at the distribution of *Equus* species in South America (Figure 1), the typical Pampean species, and index to the Lujanian SALMA, *E. neogeus,* occurs in eastern South America, but does not occur outside of lowland Argentina, Uruguay, and Brazil. The other four recognized species, including *E. insulatus*, are primarily distributed at higher elevations in the Andean and sub-Andean regions of South America. Several workers in the past have postulated multiple “ecogeographic” dispersal routes during the GABI [35]. One plausible hypothesis to reconcile this disparity in earlier occurrences outside of lowland South America is that the initial dispersal route for *Equus*, and represented by the other four species including *E. insulatus*, occurred in the Andean and sub-Andean regions (but not in the lowlands) during the Ensenadan. This likely corresponds to GABI 3 at 1.0 to 0.8 Ma [2], [3]. *Equus* was thus biogeographically restricted to higher elevations until the late Pleistocene at ∼0.125 Ma at which time *E. neogeus* first appeared in lowland South America. The most parsimonious hypothesis is that *E. neogeus* is most closely related to one of the other species of South American extinct *Equus*. Likewise, the dental morphology indicates that all of these species fit into the caballine clade [36], [37]. It is nevertheless intriguing to speculate, from a phylogeographic point of view, if *E. neogeus* may have originated independently of the other South American endemic *Equus* from a North American sister species within the caballine clade, thus suggesting a second dispersal of *Equus* during GABI 4 at 0.125 Ma. The test of these interesting scenarios must await a comprehensive phylogeographic analysis of extinct species of *Equus* from both North and South America. These studies likewise may inform, or then be compared with, recent molecular phylogenetic analyses of Pleistocene *Equus* using ancient DNA [38], [39].

### Conclusions

Given the paucity of well calibrated fossil localities outside of the classic areas in Argentina, the mammal fauna from the Tarija basin in southern Bolivia is an anchor point for our understanding of SALMA chronology during the middle Pleistocene Ensenadan SALMA. Previous studies that questioned of the occurrence of *Equus* at Tarija argued that given its imprecise biostratigraphy, it perhaps could have been restricted to the upper part of the Tolomosa Formation which is best calibrated to be <0.78 Ma (the age of the Brunhes-Matuyama boundary), but how much younger within the Pleistocene has remained uncertain. With the unambiguous documentation of *E. insulatus* occurring at all 15 biostratigraphic levels or zones in the chronostratigraphic sequence at Tarija, including three that are demonstrably older than 0.76±0.03 Ma, then the argument that the entire sequence is late Pleistocene is no longer tenable. *Equus insulatus* unambiguously occurs during the Ensenadan SALMA, as this biochron is defined from Argentina. So far as can be documented, *E. insulatus* was part of the GABI 3 (not GABI 4), which is calibrated to be ∼1.0 to 0.8 Ma [3].

The Great American Biotic Interchange (GABI) is a complex history of multiple dispersal events between the Americas during the Pliocene and Pleistocene. Further refinement of the individual taxonomic dispersals and number of immigration events during GABI will require in the future an additional level of chronostratigraphic and biostratigraphic precision not yet available, but is theoretically possible, in South America.

## Supporting Information

Table S1 **Collection database of Tarija equids (Family Equidae).** University of Florida Vertebrate Paleontology database of all relevant specimens from Tarija, Bolivia that was used as the basis for this study. The database includes taxonomic, locality, and stratigraphic data, as well as the nature of the specimen, e.g., isolated tooth and which position. For the morphological analysis presented in Figure 6, the relevant measurements are presented for all first (M1) or second (M2) upper molars.
(XLS)
